# Low-pathogenicity *Mycoplasma* spp. alter human monocyte and macrophage function and are highly prevalent among patients with ventilator-acquired pneumonia

**DOI:** 10.1136/thoraxjnl-2015-208050

**Published:** 2016-04-12

**Authors:** T J Nolan, N J Gadsby, T P Hellyer, K E Templeton, R McMullan, J P McKenna, J Rennie, C T Robb, T S Walsh, A G Rossi, A Conway Morris, A J Simpson

**Affiliations:** 1 MRC Centre for Inflammation Research, University of Edinburgh, Edinburgh, UK; 2 Clinical Microbiology, NHS Lothian, Edinburgh, UK; 3 Institute of Cellular Medicine, Newcastle University, Newcastle, UK; 4 Centre for Infection and Immunity, Queen's University, Belfast, UK; 5 Department of Microbiology, Belfast Health & Social Care Trust, Belfast, UK; 6 Division of Anaesthesia, Department of Medicine, University of Cambridge, Cambridge, UK

**Keywords:** Pneumonia, Assisted Ventilation, Innate Immunity, Bacterial Infection, Cytokine Biology

## Abstract

**Background:**

Ventilator-acquired pneumonia (VAP) remains a significant problem within intensive care units (ICUs). There is a growing recognition of the impact of critical-illness-induced immunoparesis on the pathogenesis of VAP, but the mechanisms remain incompletely understood. We hypothesised that, because of limitations in their routine detection, Mycoplasmataceae are more prevalent among patients with VAP than previously recognised, and that these organisms potentially impair immune cell function.

**Methods and setting:**

159 patients were recruited from 12 UK ICUs. All patients had suspected VAP and underwent bronchoscopy and bronchoalveolar lavage (BAL). VAP was defined as growth of organisms at >10^4^ colony forming units per ml of BAL fluid on conventional culture. Samples were tested for Mycoplasmataceae (*Mycoplasma* and *Ureaplasma* spp.) by PCR, and positive samples underwent sequencing for speciation. 36 healthy donors underwent BAL for comparison. Additionally, healthy donor monocytes and macrophages were exposed to *Mycoplasma salivarium* and their ability to respond to lipopolysaccharide and undertake phagocytosis was assessed.

**Results:**

Mycoplasmataceae were found in 49% (95% CI 33% to 65%) of patients with VAP, compared with 14% (95% CI 9% to 25%) of patients without VAP. Patients with sterile BAL fluid had a similar prevalence to healthy donor BAL fluid (10% (95% CI 4% to 20%) vs 8% (95% CI 2% to 22%)). The most common organism identified was *M. salivarium*. Blood monocytes from healthy volunteers incubated with *M. salivarium* displayed an impaired TNF-α response to lipopolysaccharide (p=0.0003), as did monocyte-derived macrophages (MDMs) (p=0.024). MDM exposed to *M. salivarium* demonstrated impaired phagocytosis (p=0.005).

**Discussion and conclusions:**

This study demonstrates a high prevalence of Mycoplasmataceae among patients with VAP, with a markedly lower prevalence among patients with suspected VAP in whom subsequent cultures refuted the diagnosis. The most common organism found, *M. salivarium*, is able to alter the functions of key immune cells. Mycoplasmataceae may contribute to VAP pathogenesis.

**Key messages**

**What is the key question?**

- What is the prevalence of Mycoplasmataceae among patients with ventilator-acquired pneumonia (VAP), and does this have any pathophysiological relevance?

**What is the bottom line?**

- Patients with VAP have a high prevalence of Mycoplasmataceae compared with similar patients without VAP, and the most common species found can impair immune cell function.

**Why read on?**

- These findings provide a novel insight into the biology of VAP and suggest new potential prevention strategies.

## Introduction

Ventilator-acquired pneumonia (VAP) remains a significant cause of morbidity and mortality in patients admitted to the intensive care unit (ICU).^1^ There is a growing recognition of failure of immune cell function (immunoparesis) in the lung^2^ and peripherally^3^ ^4^ in the pathogenesis of VAP. The mediators of this immunoparesis remain incompletely understood, although it is likely that it arises from multifactorial insults involving both host and microbial factors. It is now widely recognised that the predominant route for infection is via ‘microaspiration’ of organisms from the hypopharynx, allowing colonisation and subsequent infection of the lower airways.^1^

The organisms which cause VAP have traditionally been thought to be conventional bacterial species, such as *Staphylococcus aureus* and *Pseudomonas aeruginosa*.^5^ However our understanding of the microbiology of VAP is heavily influenced by the relative ease of microbial culture. In recent years there has been a greater understanding of the human microbiome and the influence of bacteria that are not cultured.^6^ Recent reports have suggested that non-classical organisms, such as *Mycoplasma* spp., may be present in patients with VAP.^7^ ^8^ The methods of detection used in these studies have been variable and there was little evidence of the impact these species may have on host immunity.

Mycoplasmataceae are members of the mollicutes class of bacteria, which lack a cell wall and have the smallest genomes found in self-replicating organisms.^9^ Their limited biosynthetic capability makes culture and detection problematic, needing specialised growth media and prolonged incubation or reliance on indirect methods such as serology.^9^ A range of Mycoplasmataceae such as *Mycoplasma salivarium* and *M. orale* are generally considered to be human commensals,^10^ while others such as *M. pneumoniae* and *Ureaplasma urealyticum* are recognised pathogens of the respiratory and genitourinary tract, respectively. However, even the *Mycoplasma* spp. commonly thought to be commensals have been shown to cause infections in immunocompromised hosts.^11^ Additionally, co-infection with *Mycoplasma* spp. has been implicated in accelerated progression of HIV/AIDS.^12^

We hypothesised that patients with confirmed VAP would have a higher prevalence of Mycoplasmataceae than patients without VAP, and that the dominant organism found (*M. salivarium)* would impair the ability of monocytes and macrophages to respond to further bacterial stimuli. Accordingly, this study had two aims; first, to quantify the prevalence of Mycoplasmataceae in two cohorts of patients with suspected VAP and determine which species were present. The second aim was to examine the effect of a classically non-pathogenic Mycoplasma, *M. salivarium*, on the ability of monocytes and macrophages to respond to pathogenic stimuli.

## Methods

### Patients and volunteers

We constructed the subject cohorts for this study from clinical samples and data from two recent patient studies and a healthy volunteer cohort.^13^ ^14^ The first patient cohort was recruited from two general, teaching hospital ICUs from 2005 to 2009. Patients were included if they fulfilled clinical criteria for suspected VAP, that is, mechanical ventilation for >48 h, new infiltrates on chest radiograph, and at least two of the following: temperature >38°C, leucocyte count >11×10^9^ per litre of peripheral blood or purulent tracheal secretions.

The second patient cohort was recruited from 12 ICUs across the UK from 2012 to 2013, and met similar enrolment criteria to those in patient cohort 1. Both cohorts 1 and 2 underwent a standardised bronchoscopy and bronchoalveolar lavage (BAL) as described.^13^ ^14^ In both cohorts VAP was confirmed by growth of organisms at >10^4^ colony forming units per ml of BAL fluid (CFU/mL) on conventional culture.

The healthy volunteers were recruited from the University of Edinburgh and from a primary care practice. Volunteers underwent either bronchoscopy and BAL using the same protocol as used in patient cohort 1, or phlebotomy for extraction of peripheral blood leucocytes for the leucocyte function experiments detailed below.

### Sample processing

BAL fluid was prepared as described,^13^ with the cell-free supernatant being stored at −80°C. Aliquots of BAL fluid from patient cohort 1 and healthy volunteers underwent nucleic acid extraction by one of the authors (NJG) using the DNeasy Blood and Tissue kit (Qiagen, Manchester, UK) with pretreatment protocol for Gram-positive bacteria, according to the manufacturer's instructions. Extractions for patient cohort 2 were undertaken by another author (JPM) using the MagNA pure 96 DNA and viral NA small volume kit (Roche, Indianapolis, Indiana, USA) in a geographically separate laboratory. The extraction in two laboratories was undertaken to confirm that the *Mycoplasma* detection was not occurring due to contamination of consumables in one or other laboratory. Further confirmation came by running the PCR on saline passed through the same extraction columns but without contact with the patient or volunteer BAL fluid samples.

PCR for *Mycoplasma* spp. was based on the method of van Kuppeveld *et al*^15^ targeting the 16S rRNA gene using forward primer general prokaryotic oligonucleotides-1 (GPO-1) and reverse primer mycoplasma genus-specific oligonucleotides (MGSO) and gel electrophoresis for detection. Positive extracts were sequenced on the reverse strand to determine *Mycoplasma* spp. using the QIAquick PCR purification kit (Qiagen) for purification, and ABI Prism BigDye Terminator and the ABI 3730 instrument (Applied Biosystems, Life Technologies, Paisley, UK) for Sanger sequencing. *Ureaplasma* spp. real-time PCR was based on an inhouse unpublished method targeting the *urease* gene using the following oligonucleotides; UreF: ACG WCG TTT CGA TAT TCC AT, UreR: TTC CRT TAA CTA AGC CRT TT and UreP: 6′FAM—TCG TTT TGA ACC AGG AGA YAA AA—BHQ1. Reactions comprised HotStarTaq (Qiagen) reagents, 0.5 μM primer Ure-F, 0.65 μM primer UreR, 0.2 μM probe UreP and 10 μL nucleic acid extract to a final volume of 25 μL. Real-time PCR was carried out on the ABI 7500 instrument (Applied Biosystems). *Ureaplasma* spp. positive extracts were speciated using the method of Kong *et al*^16^ targeting the 5′ end of the *MBA* gene with primers UMS-57*c* and UMA222 for *U. parvum* and UMS-170*c* and UMA263 for *U. urealyticum*, followed by gel-based detection.

### Human inflammatory cell/*Mycoplasma* interactions

Human mononuclear cells were obtained from whole blood from healthy donors using previously described methods.^17^ ^18^ Briefly, leucocytes were obtained by dextran sedimentation, with separation of mononuclear and granulocytic cells over Percoll gradients. CD14+ve monocytes were extracted from the mononuclear fraction using a magnetic bead system (MACS, Miltenyi Biotec, Bisley, UK), and >95% purity confirmed by flow cytometry. Monocytes were either used fresh at 300 000 per well, or matured into monocyte-derived macrophages (MDMs). MDMs were produced by adhering 300 000 monocytes to 24-well tissue culture plates (Corning Costar, Sigma-Aldrich, Gillingham, UK) and incubating in Iscove's modified Dulbecco's medium and 10% autologous serum for 7 days. MDM maturation was confirmed morphologically using light microscopy.

### Exposure to *Mycoplasma*

Monocytes and MDMs were initially exposed to three titres of live *M. salivarium* strain NC10113 (Mycoplasma Experience, Bletchingley, UK), namely multiplicity of infection (MOI) of 1:10 (low), 1:1 (moderate) and 10:1 (high) for 24 h at 37°C and 5% CO_2_. Cell death was assessed by nuclear staining using Sytox Green (Life Technologies) and fluorescence microscopy, counting the number of fluorescent cells in three randomly selected fields per well, with a minimum of 100 cells per field. All experiments were conducted in duplicate using a minimum of n=3 individual donors.

### Influence of *Mycoplasma* on response to lipopolysaccharide

Monocytes and MDMs were cultured as above and exposed to either live *M. salivarium* or vehicle control for 24 h. Supernatants were removed, centrifuged at 200 g for 10 min and frozen for later analysis. In subsequent experiments monocytes and MDMs were cultured with *M. salivarium* or vehicle control for 24 h and then, without washing or changing supernatant, exposed to lipopolysaccharide (*Escherichia coli* 0127:B8, Sigma-Aldrich, Gillingham, UK) at 100 ng/mL for a further 24 h, before supernatants were removed and processed as above. Cytokines were assayed using Cytometric Bead Array (BD Biosciences, Oxford, UK).

### Effect of *Mycoplasma* on phagocytosis by MDMs

MDMs were exposed to live *M. salivarium* or vehicle control for either short (60 min) or long (24 h) incubations. Following this the cells were presented with a phagocytic target, pHrodo Red Zymosan (Life Technologies) that had been opsonised in autologous donor serum. Cells were exposed to 0.02 mg/mL zymosan for 30 min before being placed on ice and phagocytosis assessed by fluorescence microscopy.

### Statistical analysis

Categorical variables were expressed as proportions and compared using Fisher's exact test. Continuous variables were expressed as mean values and compared using one-way analysis of variance (ANOVA) with Bonferroni's post hoc test for comparison between individual conditions. All analyses were performed using Prism (Graphpad Software, La Jolla, California, USA). p≤0.05 was considered statistically significant.

### Ethical approval

The samples from patient cohorts 1 and 2 were collected in studies approved by the Lothian Research Ethics Committee (REC) (LREC/2002/8/19) and NRES North East REC (11/NE/0242) and Scotland A REC (11/SS/0089), respectively, with the informed consent/assent from the next of kin. The healthy volunteer BAL fluid was obtained from volunteers in a study approved by Lothian REC (06/S1101/50) with all volunteers giving written informed consent. The monocyte and MDM experiments were conducted using cells obtained under the authorisation of the Lothian REC (08/S1103/38) following written, informed consent.

## Results

### Settings and patients

In total there were 159 patients (67 from cohort 1, 92 from cohort 2) for analysis, of whom 43 (13 from cohort 1 and 30 from cohort 2) had confirmed VAP. The demographics of patient cohorts 1 and 2 have been described previously;^13^ ^14^ briefly the patients were 69% male, with a median age of 60 years (IQR 48–71), 45% were admitted to ICU with surgical diagnoses with the remainder admitted with medical diagnoses. Median APACHE II score was 20 (IQR 16–24), ICU mortality was 28% and hospital mortality 35%. Further demographic details are shown in the online supplementary table S1. Thirty-six healthy donors underwent BAL using the same protocol as cohort 1.^13^

10.1136/thoraxjnl-2015-208050.supp1 Supplementary data

### Detection of *Mycoplasma* and *Ureaplasma* spp.

**Figure 1:**
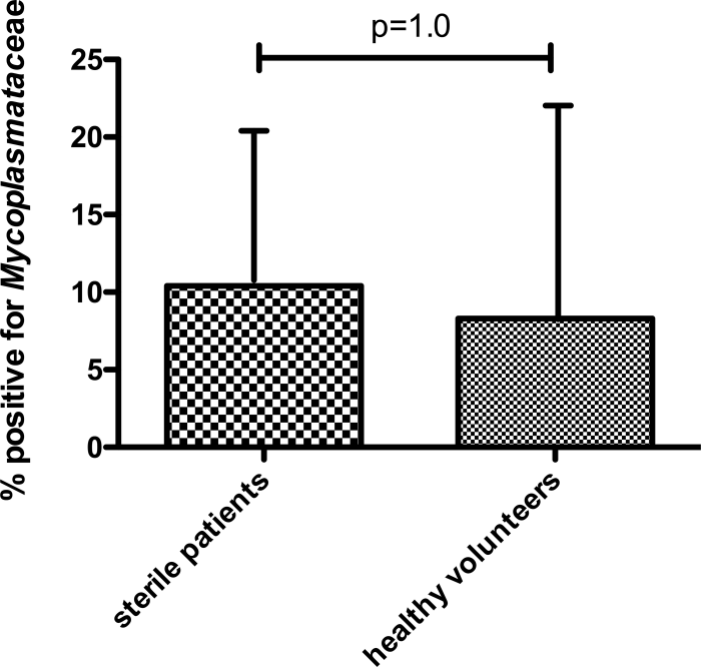
Proportion of healthy volunteers and patients with no microbial growth (by conventional culture) in whom Mycoplasmataceae were detected. Data shown as proportion and upper 95% CI. p Value by Fisher's exact test.

Detection of Mycoplasmataceae (*Mycoplasma* and *Ureaplasma* spp.) occurred significantly more frequently in patients with VAP compared with those without VAP (table 1). This was the case for both patient cohorts. Among patients without VAP, the frequency of Mycoplasmataceae was higher in those who had some growth on culture as opposed to those whose BAL fluid was sterile by conventional culture, although none of these differences reached statistical significance (table 1). The frequency of Mycoplasmataceae detection did not differ significantly between patients with sterile cultures and healthy volunteers (p=1.0 by Fisher's exact test, figure 1), nor was the trend in difference between all non-VAP patients and healthy volunteers significant (14% (95% CI 9% to 25%) vs 8% (95% CI 2% to 22%), p=0.57 by Fisher's exact test).

**Table 1:** Frequency of Mycoplasmataceae detection in patients with and without VAP confirmed by conventional microbiological culture

			Non-VAP subdivision	
Cohort	VAP (>10^4^ CFU/mL)	Non-VAP (<10^4^ CFU/mL or sterile)	Sub-10^4^ CFU/mL growth	Sterile
1	7/13 54% (25–81%)	10/54 19% (9–31%) (p=0.014)	6/23 26% (10–48%)	4/31 13% (4–31%) (p=0.29)
2	14/30 47% (28–66%)	6/62 10% (4–20%) (p=0.0001)	3/26 12% (2–30%)	3/36 8% (2–22%) (p=0.69)
Combined cohorts	21/43 49% (33–65%)	16/116 14% (9–25%) (p<0.0001)	9/49 18% (9–32%)	7/67 10% (4–20%) (p=0.28)

The non-VAP subdivision is of patients with growth below the 10^4^ CFU/mL cut-off and those with no detectable growth**.** Data are shown as raw values, percentage (95% CI by Clopper–Pearson method), p values by Fisher's exact test for comparison between VAP and non-VAP groups (column 3) and for comparison between sub-10^4^ CFU/mL growth and sterile (column 5).

CFU, colony forming unit; VAP, ventilator-acquired pneumonia.

The majority (82%) of Mycoplasmataceae detected in patients were *Mycoplasma* spp., and within this group *M. salivarium* was the dominant organism detected (table 2). Two healthy volunteers were positive for *Mycoplasma* spp. by PCR but sequencing revealed non-Mycoplasmataceae organisms (*Solobacterium moorei*) and these samples were excluded from the ‘positive’ samples. Among the remaining three healthy volunteer samples that were positive for *Mycoplasma* spp. sequencing revealed one *M. salivarium* and two mixed species.

**Table 2:** Species of atypical organism detected in patients (two patients had both *Mycoplasma* spp. and *Ureaplasma* spp. detected)

Organism	n/frequency
*Mycoplasma hominis*	2 (5%)
*Mycoplasma salivarium*	22 (56%)
*Ureaplasma parvum*	1 (3%)
*Ureaplasma urealyticum*	4 (10%)
Mixed species of *Mycoplasma,* unable to further speciate	8 (21%)
Unable to speciate (*Ureaplasma* sp +ve by PCR)	2 (5%)

The presence of atypical organisms did not differ significantly between patients with VAP arising from differing aetiological classes (table 3).

**Table 3:** Mycoplasmataceae detection among patients with VAP (ie, growth at >10^4^CFU/mL) of various aetiologies

VAP pathogen	N=	Proportion positive for atypical organisms, % (n)
Gram-positive bacteria	12	58 (7)
Gram-negative bacteria	20	35 (7)
Fungi and yeasts	7	57 (4)
Mixed organism types	4	75 (3)

p=0.41 by χ^2^. Details of species grown are shown in online supplementary table S2.

CFU, colony forming unit; VAP, ventilator-acquired pneumonia.

### Effect of *M. salivarium* coculture on monocyte and MDM function

Following the discovery of a high prevalence of *M. salivarium* among patients with VAP (52% of Mycoplasmataceae in patients with VAP were *M. salivarium*), we hypothesised that this species may have a role to play in the pathogenesis of VAP. We therefore examined the effect of *M. salivarium* infection on the ability of key immune cells, monocytes and macrophages, to respond to bacterial stimuli.

### *M. salivarium* and cellular toxicity

**Figure 2:**
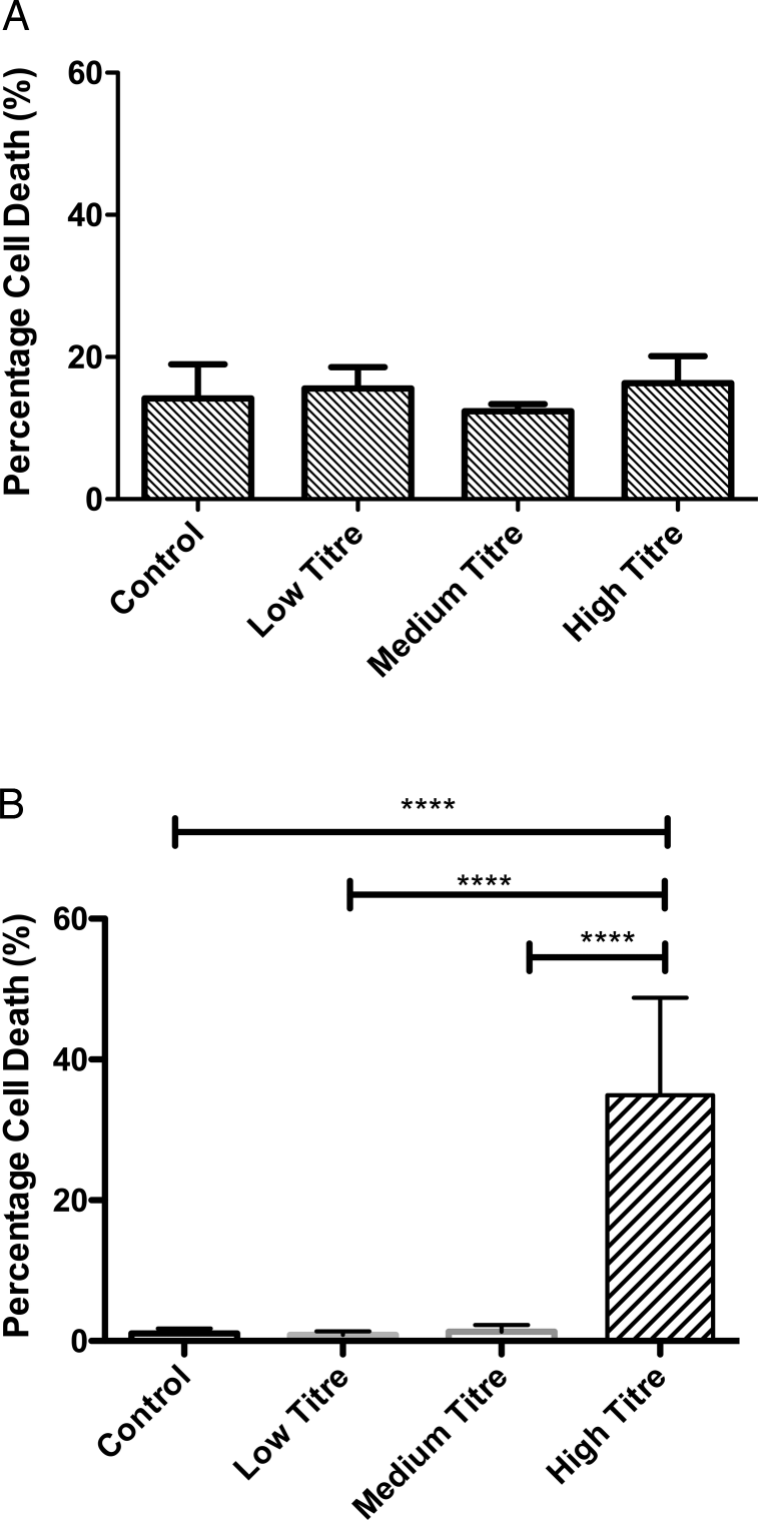
Cell death associated with *Mycoplasma salivarium* in monocytes and monocyte-derived macrophages (MDMs). (A). Numbers of Sytox green positive monocytes as a proportion of total cells from three randomly selected fields per condition, n=3 individual donors. p=0.56 by ANOVA. Data shown as mean and SD. (B). Numbers of Sytox green positive MDMs as a proportion of total cells from three randomly selected fields per condition, n=3 individual donors. p<0.0001 by ANOVA, ****p<0.0001 by Bonferroni's post hoc test, all other comparisons non-significant by Bonferroni's test. Data shown as mean and SD.

Neither monocytes nor MDMs exposed to live *M. salivarium* for 24 h at MOIs of 1:10 (low) and 1:1 (moderate) exhibited significantly greater cell death than untreated cells (figure 2A, B). A high MOI (10:1) induced 30% cell death in MDMs (figure 2B, far right column). Monocytes appeared relatively resistant to such effects (figure 2A), although demonstrated an overall higher basal level of cellular death than MDMs. Subsequent experiments were confined to low and moderate exposures for both cell types.

### *M. salivarium* selectively impairs the ability of immune cells to respond to stimulation with lipopolysaccharide

**Figure 3:**
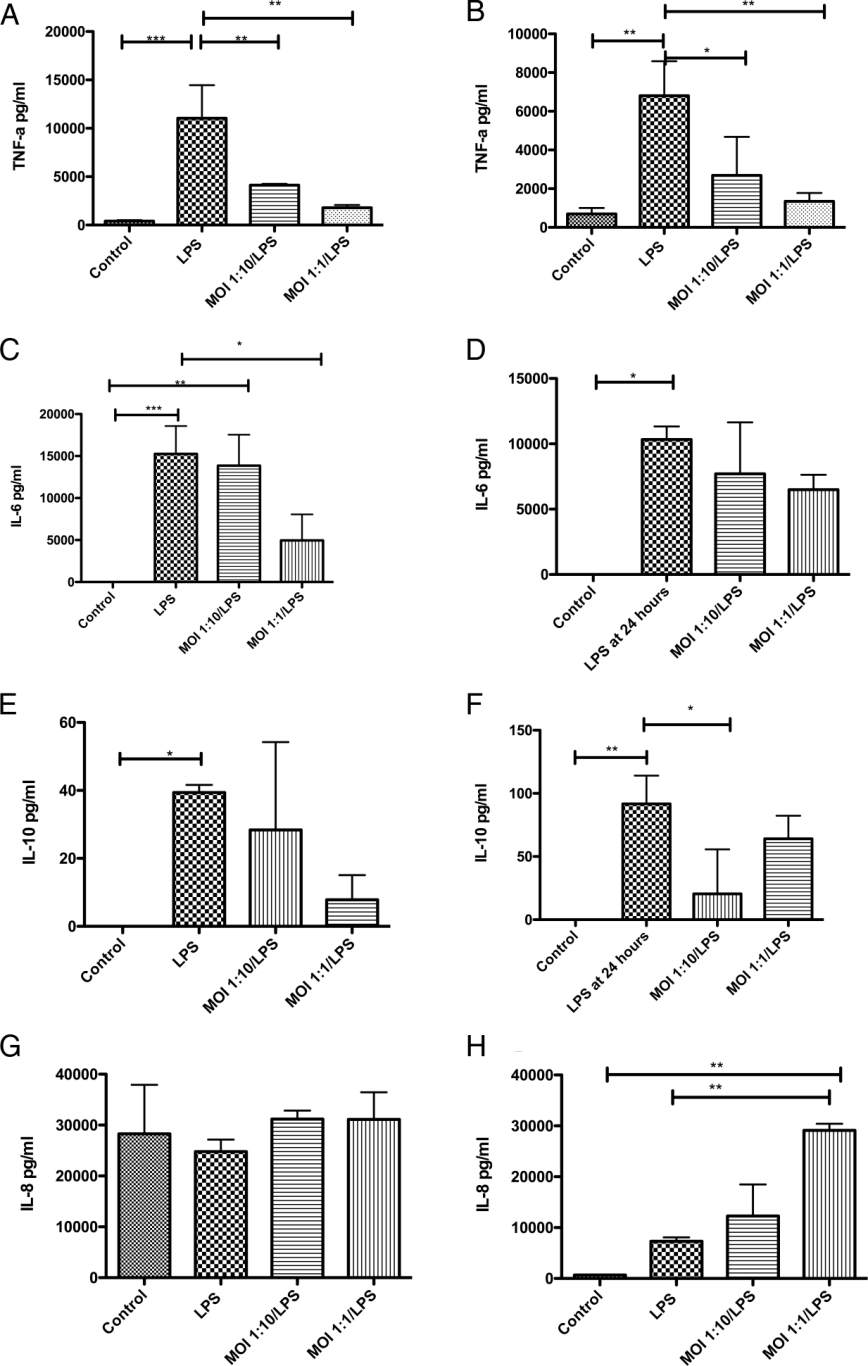
Effect of exposing monocytes and monocyte-derived macrophages (MDMs) to *Mycoplasma salivarium* and lipopolysaccharide (LPS), on cytokine secretion. (A) TNF-α secretion by monocytes, p=0.0003 by ANOVA. (B) TNF-α secretion by MDMs, p=0.0024 by ANOVA. (C) Interleukin (IL) 6 secretion by monocytes, p=0.0006 by ANOVA. (D) IL-6 secretion by MDMs, p=0.04 by ANOVA. (E) IL-10 secretion by monocytes, p=0.02 by ANOVA. (F) IL-10 secretion by MDMs, p=0.004 by ANOVA. (G) IL-8 secretion by monocytes, p=0.55 by ANOVA. (H) IL-8 secretion by MDMs, p=0.0013 by ANOVA. *p<0.05, **p<0.01, ***p<0.001 by Bonferroni's post-hoc test, all other comparisons were non-significant by Bonferroni's test. Results are from duplicates of n=3 individual healthy donors, with 300 000 cells per well. Data shown as mean and SD. MOI, multiplicity of infection.

As expected, lipopolysaccharide (LPS) alone significantly increased the secretion of tumour necrosis factor (TNF)-α, interleukin (IL) 6 and IL-10 from monocytes and MDM (figure 3); in general *M. salivarium* alone had a similar but smaller effect (see online supplementary tables S3 and S4). However, the TNF-α response to an LPS challenge was impaired following both low and moderate *M. salivarium* exposure in monocytes and macrophages (p=0.0003 and p=0.0024 by ANOVA, respectively) (figure 3A, B). A similar although slightly less pronounced pattern was seen with IL-6 (p=0.0006 and p=0.04) and IL-10 (p=0.02 and p=0.004) secretion (figure 3C–F). IL-8 secretion showed a contrasting pattern, being unchanged in monocytes (p=0.55) while macrophages showed enhanced IL-8 secretion with *Mycoplasma* exposure (p=0.0013) (figure 3G, H).

### *M. salivarium* impairs phagocytosis by macrophages

**Figure 4:**
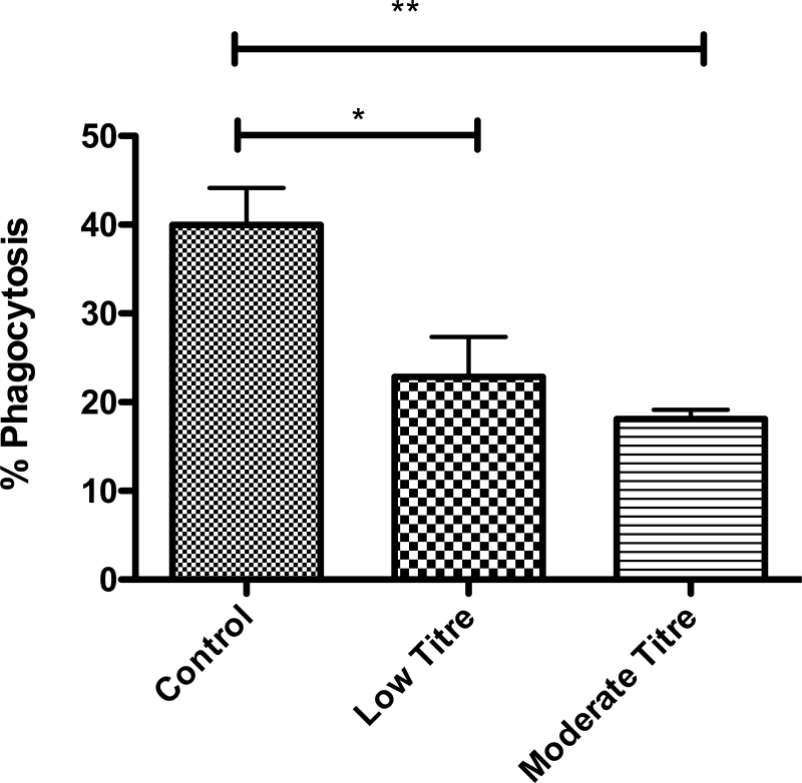
Phagocytosis of zymosan by monocyte-derived macrophages (MDMs) exposed to *Mycoplasma salivarium*. 24 h exposure at multiplicity of infection (MOI) of 1:10 (low) and 1:1 (moderate) titre. p=0.005 by ANOVA. Data shown as mean and SD. *p<0.05, **p<0.01 by Bonferroni's post-hoc test, all other comparisons non-significant. Results are from duplicates of n=4 individual healthy donors, with 300 000 cells per well, data shown as mean and 95% CI.

Phagocytosis of microbes and other particulate matter by macrophages is a key part of pulmonary defences, as it directs destruction of the microorganism and facilitates antigen presentation to effector cells^19^ *M. salivarium* significantly impaired phagocytosis by macrophages following 24 h exposure (figure 4), with moderate titre having a more pronounced effect than low titre (p=0.005 by ANOVA).

## Discussion

This study is, to our knowledge, the most comprehensive description of Mycoplasmataceae in VAP to date. Through the use of molecular diagnostics we have confirmed previous findings of *Mycoplasma* presence^7^ ^8^ in a more robust manner and, importantly, identified the species involved. We have also demonstrated the presence of small numbers of *Ureaplasma* spp., organisms more commonly found in genitourinary tract infections.

It is, perhaps, surprising that the dominant organism is an oral commensal which is commonly regarded as non-pathogenic.^10^ We did not find a single classically pathogenic *Mycoplasma* spp., which is in contrast to findings from authors in other countries.^7^ ^8^ However *M. salivarium* is found in the oral cavity of 97% of the healthy population,^10^ and therefore is available to be microaspirated past the endotracheal cuff alongside other organisms in the oropharyngeal space.^5^ Although we undertook targeted PCR rather than pan-bacterial metagenomics, the appearance of Mycoplasmataceae may reflect a change in the lung microbiome, an effect that has been noted in other pulmonary infections.^20^ The role of lung microbiome shift in the pathogenesis and maintenance of pulmonary infection is an area of active, ongoing investigation.^20^

The striking difference in prevalence between patients with and without microbiologically confirmed VAP invites several possible explanations. First, the presence of *Mycoplasma* spp. in BAL may simply reflect the burden of microaspiration from the oropharynx. Second, the presence of *Mycoplasma* in BAL fluid may be indicative of a relative immune failure^2^ which leaves patients unable to clear these low pathogenicity organisms, with the immune failure being more pronounced in those who develop VAP. A third possibility is that *Mycoplasma* may contribute to the pathogenesis of VAP by directly impairing immune function, thus facilitating infection by other ‘classical’ VAP pathogens.

Although we cannot answer these questions definitively, and there may be contributions from all three, we undertook the monocyte/*Mycoplasma* experiments to look for proof of principle of *Mycoplasma*-induced immune impairment that may support the third hypothesis. Among patients with HIV/AIDS, co-infection with *Mycoplasma* spp. is associated with faster progression of a disease which is characterised by immune failure,^12^ although the causative relationship remains controversial^21^ ^22^ and the *Mycoplasma* spp. implicated are those more commonly associated with the genitourinary tract.^21^ In previous in vitro studies in human monocytic cell lines^23^ and bovine monocytes^24^ the presence of unspecified *Mycoplasma* spp.^23^ and *M. bovis*^24^ respectively have been associated with impaired immune responses. The mechanism by which *M. salivarium* alters innate immune cell function is the focus of ongoing work, however our data do not support this being simply an effect of cellular toxicity. Interestingly, the effects on cytokine secretion are complex with reductions in LPS-induced secretion of TNF-α, IL-6 and IL-10 but unaffected (monocyte) or stimulated (MDM) IL-8 production. The standard description of ‘deactivated/reprogrammed’ monocytes in patients with sepsis is of reduced TNF-α and enhanced IL-10 secretion.^25^ Thus the effects we have seen with *Mycoplasma* may reflect a related but different phenomenon to classical ‘LPS tolerance’.^25^ Indeed Zakharova *et al* found a similar *Mycoplasma*-mediated suppression of IL-10 transcription in monocyte-like THP-1 cells.^23^ Although we can only speculate on the potential in vivo effects, it is possible that when *Mycoplasma* spp. enter the already inflamed lung their predominant suppressive effect is maladaptive and promotes infection but the same effect in its ‘normal’ environment contributes to an adaptive microbiota-induced immune tolerance.^20^ ^26^

We believe this study has a number of strengths. First we examined a large number of samples taken from the alveolar region of the lungs of patients with suspected VAP and these samples came from a range of locations throughout the UK. It is therefore unlikely that these findings are specific to a single unit or geographical region. Second, by using the latest molecular techniques we were able (A) to identify *Mycoplasma* with greater confidence than previous studies which relied on *Mycoplasma* toxin detection^7^ or limited PCR,^8^ and (B) to identify the specific species involved. Third, by undertaking examination of the effects of the most common identified species, *M. salivarium*, on two key innate immune cells, we were able to strengthen our hypothesis that this organism is not simply a bystander but is contributing to the immunoparesis that facilitates VAP.

Our study is limited by having a sample from a single time point, at the time of suspected infection. Therefore we cannot comment on when the Mycoplasmataceae appeared in the alveolar space relative to the development of VAP. As such we are certainly unable to infer a causative relationship between Mycoplasmataceae and subsequent VAP. Our positive event rate of 21 patients with Mycoplasmataceae and VAP prevents more in-depth examination of the relationship between Mycoplasmataceae, clinical features and outcomes from VAP. We were also unable to precisely quantify the amounts of non-classical organisms present in the lungs of patients, although the ability to obtain good speciation data for the great majority of the *Mycoplasma* positive samples from patients with VAP suggests a reasonable bacterial load. The titres *of M. salivarium* to which the monocytic cells were exposed therefore remain an approximation of the situation in vivo. Indeed our in vitro model of *Mycoplasma* infection is necessarily a simplification of the in vivo situation.

The implications of this work are that preventing lower respiratory tract *Mycoplasma* spp. infection, through improved oral hygiene measures and prevention of microaspiration, may contribute to preventing VAP through immune restoration. This hypothesis could also provide a novel explanation for the finding that incorporating macrolides to antibiotics for VAP improves outcomes,^27^ beyond the more widely accepted immunomodulatory effects of macrolides.^28^ We do not, however, advocate broadening antibiotic coverage on the basis of our findings, as only further clinical trials could justify such an approach. The high prevalence of Mycoplasmataceae among patients with VAP should also be considered when pan-bacterial molecular diagnostics, such as 16S rRNA gene PCR, are being used for pathogen detection in VAP; mixed sequences are difficult to resolve using conventional Sanger-based sequencing and may require next-generation sequencing methodologies.

In conclusion we have found a high prevalence of non-classical bacterial species among patients with VAP, specifically organisms such as *M. salivarium* that are traditionally thought to be non-pathogenic. We have further demonstrated that these organisms can alter antibacterial functions in healthy volunteer monocytes and MDMs, and may have the potential to contribute to the immunoparesis seen in critically ill patients.
