# Supplementary material for: Low-pathogenicity Mycoplasma spp. alter human monocyte and macrophage function and are highly prevalent among patients with ventilator-acquired pneumonia
Source: Thorax. 2016 Apr 12;71(7):594–600. doi: 10.1136/thoraxjnl-2015-208050 (PMC4941152; doi:10.1136/thoraxjnl-2015-208050)
Supplement: Supplementary data [file thoraxjnl-2015-208050supp_tables.pdf]

Demographic feature	VAP	Non-VAP	*Mycoplasmataceae* +	*Mycoplasmataceae* -
Age, years (median, IQR)	62 (49-72)	58 (48-69)	55 (34-62)	62 (49-73)
% male	77%	66%	83%	64%
Admission APACHE II score (median, IQR)	20 (16-24)	20 (16-24)	18 (15-23)	21 (16-25)
ICU length of stay (median, IQR)	24 (17-34)	21 (12-31)	24 (14-34)	21 (13-32)
ICU mortality	20% (9-36%)	31% (20-40%)	9% (2-24%)	33% (25%-42%)

Table S1: Clinical and demographic features of patients (from both cohort 1 and 2) divided by those with and without VAP, and those with and without detectable *Mycoplasmataceae*. IQR, inter-quartile range.

Organism	*Myco* +ve VAP (>10^4 CFU/ml)	*Myco* –ve VAP (>10^4 CFU/ml)	*Myco* +ve non-VAP (<10^4/CFU/ml)	*Myco* –ve non-VAP (<10^4/CFU/ml)
Escherichia coli	4	3	0	0
Enterobacter cloacae	1	1	0	6
Klebsiella pneumoniae	1	1	1	5
Proteus mirabilis	1	1	0	
Citrobacter koseri	1	1	0	
Pseudomonas aeruginosa	1	3	1	4
Coliform (not further identified)	1	0	0	1
Acinetobacter baumanii	0	1	0	1
Haemophilus parainfluenzae	0	2	2	0
Hafnia alvei	0	0	0	1
Moraxella catarrhalis	0	1	0	
Pantoea spp.	0	0	0	1
Serratia marcescens	0	0	0	1
Enterococcus faecium	0	0	0	1
Staphylococcus aureus	9 (3 MRSA)	4 (0 MRSA)	3 (1 MRSA)	8 (4 MRSA)
Coagulase negative *Staphylococcus*	0	1	2	2
Streptococcus spp.	1	1	0	2
Anaerobes	0	0	1	0
Aspergillus fumigatus	0	1	0	0
Candida spp.	4	2	2	13
No growth	0	0	7	60

Table S2: Numbers of species identified in VAP and non-VAP patients with and without detectable *Mycoplasmataceae* (denoted by *Myco* in top row). Some patients grew more than one organism. MRSA=methicillin-resistant *Staphylococcus aureus*.

Conditions/ Cytokine	Monos +control	Monos + low titre	Monos + moderate titre	P value by ANOVA
TNF-α	396 (103)	5727 (614)	4737 (3804)	0.06
IL-6	16 (13)	23350 (947) ***	4931 (2781) *	0.0001
IL-10	0 (0)	65 (56)	22 (19)	0.14
IL-8	28244 (9641)	6918 (1869)*	27885 (6853)	0.014

Table S3: Cytokine concentrations in supernatants from monocytes exposed to vehicle control, *Mycoplasma salivarium* (in low titre or medium titre) or LPS for 24 hours. Data shown as mean and standard deviation; all values are expressed in pg/ml. *P<0.05, ***P<0.001 by Bonferroni's post-hoc test, for comparison with control conditions, where no p value indicated then P is >0.05

Conditions/ Cytokine	MDM +control	MDM + low titre	MDM + moderate titre	P value by ANOVA
TNF-α	698 (309)	1857 (929)	2149 (825)	0.12
IL-6	0(0)	348 (339)	4363 (2515)*	0.02
IL-10	0 (0)	0 (0)	88 (32)**	0.002
IL-8	677 (144)	8377 (6363)	19551 (14360)	0.18

Table S4: Cytokine concentrations in supernatants from MDM exposed to vehicle control, My*coplasma salivarium* (in low titre or medium titre) or LPS for 24 hours. Data shown as mean and standard deviation; all values are expressed in pg/ml. *P<0.05, ** P<0.01, by Bonferroni's post-hoc test, for comparison with control conditions, where no p value indicated then P is >0.05
